# Data on microbial DNA-induced IL-1β production in monocytes of type 1 diabetes patients

**DOI:** 10.1016/j.dib.2019.104321

**Published:** 2019-07-27

**Authors:** Irena Zentsova, Zuzana Parackova, Jana Kayserova, Lenka Palova-Jelinkova, Petra Vrabcova, Nikol Volfova, Zdenek Sumnik, Stepanka Pruhova, Lenka Petruzelkova, Anna Sediva

**Affiliations:** a Department of Immunology, 2nd Faculty of Medicine, Charles University, University Hospital in Motol, Prague, Czech Republic; b Department of Pediatrics, 2nd Faculty of Medicine, Charles University, University Hospital in Motol, Prague, Czech Republic; c Department of Pediatrics and Adolescent Medicine, 1st Faculty of Medicine, Charles University and General University Hospital, Prague, Czech Republic; d Sotio a.c., Prague, Czech Republic

**Keywords:** Type 1 diabetes, Monocytes, DNA, Inflammasomes, Glybenclamide, NLRP3

## Abstract

Inflammasomes are large protein complexes involved in the maturation of IL-1β, a cytokine associated with the pathophysiology of type 1 diabetes (T1D). The data presented in this article focused on the role of inflammasomes in DNA recognition in T1D patients. This data extend knowledge on DNA sensing in T1D patients and relate to our research paper “Monocytes contribute to DNA sensing through the TBK1 signaling pathway in type 1 diabetes patients” Zentsova et al., 2009. To examine inflammasome involvement, we blocked the known mechanism of inflammasome activation – potassium efflux via various approaches: 1) high concentration of KCl; 2) Glybenclamide, which selectively blocks the ATP sensitive K+ channel; 3) KN-62, an inhibitor of P2X7 receptor, which activates K+ channel after ATP binding. Moreover, we used an inhibitor which blocks Nod-like receptor family containing pyrin domain 3 (NLRP3) inflammasome. In T1D patients, we show that secretion of cytokines IL-1β, TNFα, IL-6 and IFNα after microbial DNA stimulation is promoted by potassium efflux and is not dependent on P2X7 receptor signaling. Surprisingly, the microbial DNA induced IL-1β release was independent of NLRP3.

**Table:** Specifications table

Subject	Immunology and Microbiology
Specific subject area	NLRP3 independent response of DNA in monocytes by T1D patients
Type of data	Two graphs (Figures)
How data were acquired	Concentration of cytokines in supernatants was acquired by multiplex Luminex cytokine bead-based immunoassays using a Luminex-100 system (Luminex, Austin, TX). Intracellular production of cytokines in monocytes was acquired using Aria II flow cytometer (BD Biosciences) and analyzed with FlowJo software (TreeStar)
Data format	Primary data, quantified and analyzed graphs
Parameters for data collection	For detection of intracellular cytokine expression and release of cytokines in supernatants, peripheral blood mononuclear cells (PBMCs) were stimulated with synthetic CpG2216 (Invivogen) or lipopolysaccharide (Sigma Aldrich) from *Escherichia coli*. For inhibition assays, cells were pre-treated with inhibitors for 30 minutes before CpG DNA stimulation.
Description of data collection	Intracellular cytokine detection was performed using flow-cytometry staining (FACS). Cytokines were stained using IL-1β-PE (ThermoFisher) and the data were collected using Aria II flow cytometer (BD). The levels of IL-1β, IL-6, TNFα and IFNα in the cell supernatants of PBMCs were measured by multiplex Luminex cytokine bead-based immunoassays.
Data source location	Department of Immunology, 2nd Faculty of Medicine, Charles University, University Hospital in Motol Prague Czech Republic
Data accessibility	With the article
Related research article	Monocytes contribute to DNA sensing through the TBK1 signaling pathway in type 1 diabetes patients Irena Zentsova, Zuzana Parackova, Jana Kayserova, Lenka Palova-Jelinkova, Petra Vrabcova, Nikol Volfová, Zdenek Sumnik, Stepanka Pruhova, Lenka Petruzelkova, Anna Sediva Journal of Autoimmunity https://doi.org/10.1016/j.jaut.2019.06.005

**Table:** 

**Value of the data** • Extended analyses of IL-1β signaling could help to find the efficient proinflammatory cytokine blockade strategies not only for T1D patients, but also for other patients with other autoimmune diseases • These data pointed to relatively underappreciated role of glibeclamide, which is used for decreasing blood glucose levels, but not for its anti-inflammatory effect • Our data could inspire others to investigate IL-1β signaling pathway in T1D or other autoimmune diseases

## 1 Data

**Fig. 1:**
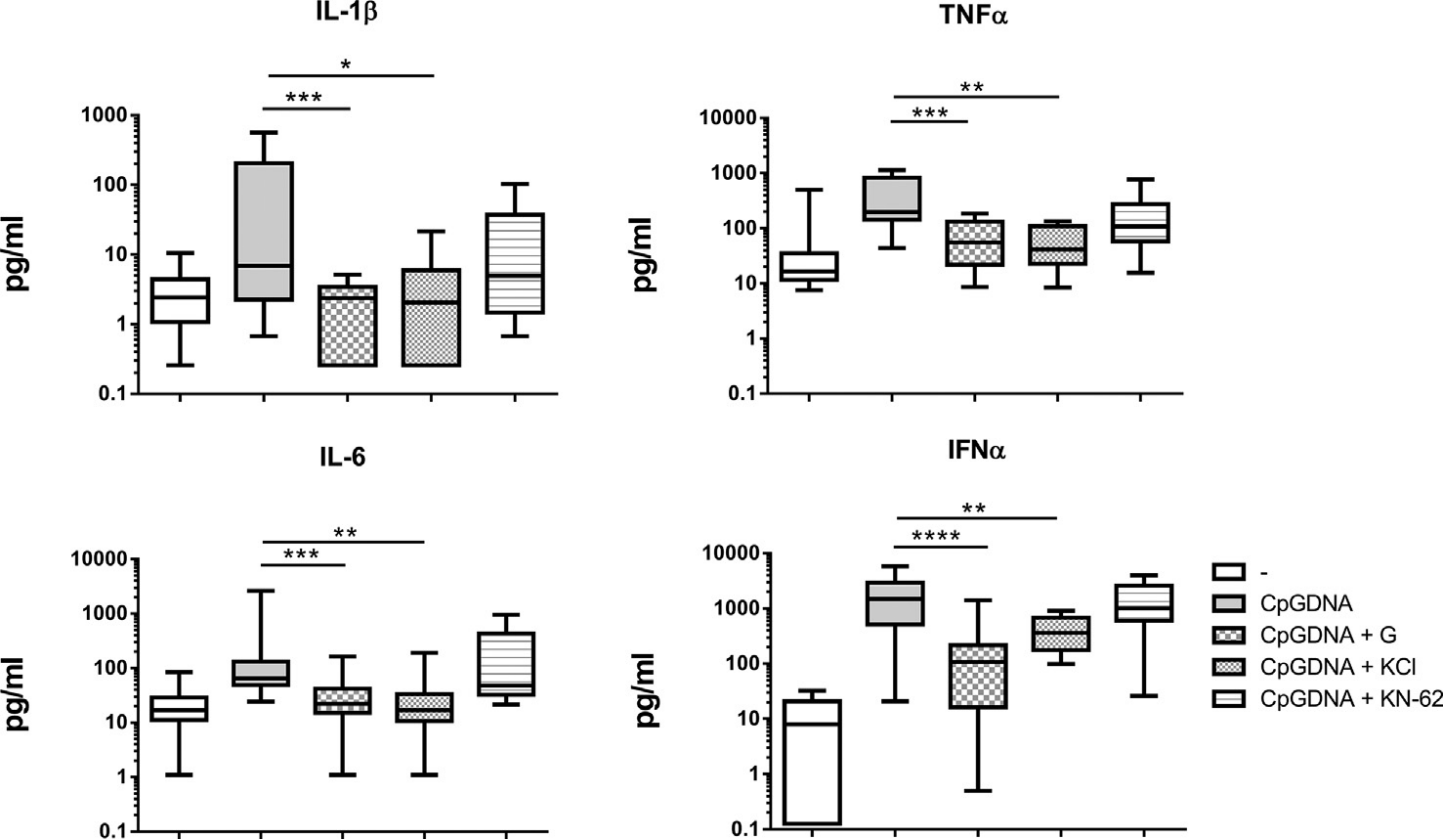
Extracellular release of cytokines by PBMCs in TID patients after CpG DNA stimulation with or without inhibition: 100μM Glibenclamide (n=13) (G), 50mM KCI (n=10), 10μM KN-62 (n=10) assessed by Luminex assay. Statistical analysis was performed using a two-tailed Wilcoxon paired *t* test. Values of p<0.05 (*), p<0.01(**) and p<0.001(***) were considered statistically significant.

**Fig. 2:**
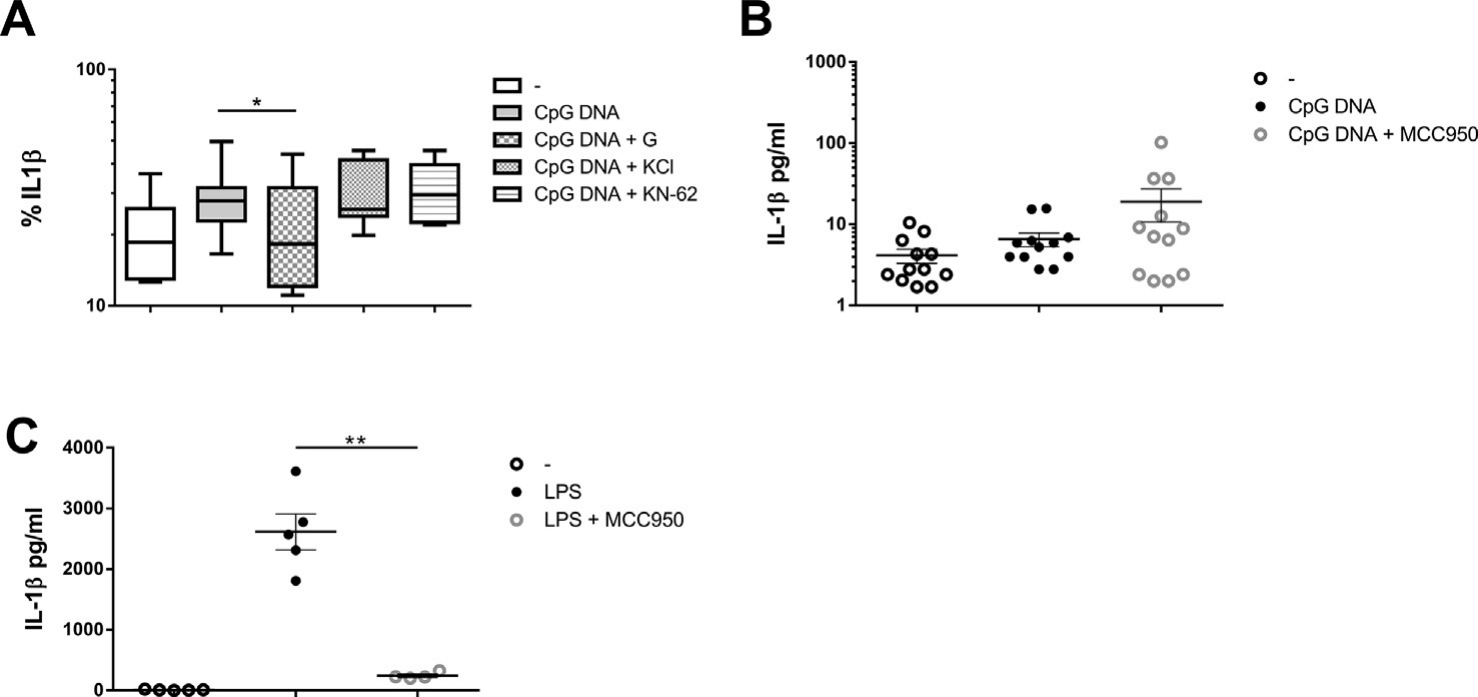
(A) Intracellular production of 1L-1β in monocytes (n=10) patients after CpG DNA stimulation with or without inhibitions: 100μM Glibenclamide (G), 50mM KCI, 10μM KN-62, as assessed by flow cytometry. (B) Production of 1L-1β in PBMCs after stimulation with CpG DNA with or without the NLRP3 inhibitor MCC950, (C) Production of 1L-1β in PBMCs pretreated with NLRP3 inhibitor MCC950 and stimulated with LPS, Statistical analysis was performed using a two-tailed Wilcoxon paried *t* test. Values of p<0.05(*) and p<0.01(**) were considered statistically significant.

As we detected in our associated study, T1D patients‘cells produce a robust proinflammatory release of cytokines after microbial DNA sensing in comparison to healthy subjects. In contrast to other cytokines, production of IL-1β was independent on TBK1 molecule [1]. This data further extend this investigation with study of IL-1β release. We described in this data article the involvement of inflammasomes, the multiprotein complexes that contain NLR (NOD like receptor) family members, in microbial DNA sensing (CpG DNA) by T1D patients. It is known, that IL-1β, which is produced as an inactive precursor proIL-1β, gets cleaved upon stimulation to a mature form via inflammasomes, especially via NLRP3 or NLRP1 [2]. We measured the release of this cytokine together with TNFα, IL-6 and IFNα in cell free supernatants of PBMCs after stimulation with CpG DNA by Luminex bead assay **(**Fig. 1**)** and studied if CpG DNA-induced cytokine release involves the potassium efflux from cells, which is a known mechanism in activation of inflammasomes [3]. PBMCs were exposed to a medium containing high concentrations of potassium chloride (50mM KCl), which prevented K+ efflux, or 100 μM Glybenclamide, which selectively blocks the ATP sensitive K+ channel, or 10 μM KN-62, an inhibitor of P2X7 receptor, which activates K+ channel after ATP binding. Glybenclamide binds to SUR1 subunits on ATP-sensitive K+ channel in pancreatic β islet cells, triggers insulin release from β cells and causes hypoglycemia in animal models, therefore glybenclamide is wildly use in patients with type II diabetes mellitus [4]. However, glybenclamide was previously used as an antimicrobial drug, and recently, besides its hypoglycemic effects, a new role for glybenclamide is suggested in regulation of inflammation [5], [6]. On T1D animal model, glybenclamide was able to prevent disease onset [7]. Our data show, that PBMCs from T1D patients pre-treated with inhibitor glybenclamide and high concentrations of KCl produced significantly less proinflammatory cytokines after stimulation with microbial CpG DNA. Pre-treatment with inhibitor KN-62 led to no change in cytokine production. Glybenclamide anti-inflammatory effect during CpG DNA stimulation was also confirmed by flow cytometry analysis Fig. 2A. Our data suggest, that the release of IL-1β and also other cytokines is dependent predominantly on K+ efflux. To further investigate the participation of NLRP3 inflammasome in the signaling, we used its inhibitor (MCC950). While the preincubation with MCC950 abolished the control LPS- induced IL-1β release Fig. 2C**,** blocking of NLRP3 before CpG DNA stimulation not affected IL-1 β production Fig. 2B**.**

## 2 Experimental design, materials, and methods

### 2.1 Patients

A long–term treated patients with T1D were included in the study. Detail characterization of patients is described in our associated study.

Written informed consent was obtained from all of the patients or the patients’ parents/guardians in accordance with the Declaration of Helsinki, and the study was approved by the Ethics Committee of University Hospital Motol.

### 2.2 Isolation of PBMCs

Peripheral blood was collected from patients into EDTA-coated tubes. First, peripheral blood mononuclear cells (PBMCs) were isolated using Ficoll-Paque (GE Healthcare Bio-Sciences, Uppsala, Sweden). The obtained cells were resuspended in RPMI 1640 medium supplemented with 2% autologous serum, 1% penicillin and streptomycin and 1% glutamax (ThermoFisher Scientific, Waltham, MA USA).

### 2.3 Detection of cytokines

For intracellular cytokine detection, PBMCs were cultured in a 24-well microtiter plate at a concentration of 1 × 10e^6^/ml and stimulated using 5 μg/ml CpG2216 (Invivogen, San Diego, CA) for 8 h in the presence of 1 μl/ml Brefeldin A (BD Biosciences, San Jose, CA, USA).

Immunophenotyping of monocytes was performed using Lin-FITC (CD3, CD19, CD20 and CD56), CD16-AlexaFluor 700, CD11c-APC, CD14-PE-DyLight594 (Exbio), HLA-DR-PerCP (BD), CD1c-BV510, CD141-BV421, and CD123-PE-Cy7 (Biolegend). IL-1β was stained using IL-1β-PE (Thermo Fisher) and the data were collected using Aria II flow cytometer (BD) and analyzed by FlowJo (TreeStar).

For extracellular cytokine detection, PBMCs were cultured in a 96-well microtiter plate at a concentration of 1 × 10e^6^/ml. The levels of IL-1β, IL-6, TNFα and IFNα in the supernatants of PBMCs were determined 24 h after the addition of 5 μg/ml CpG2216 (Invivogen, San Diego, CA) or 1 μg/ml LPS (Sigma Aldrich, St. Louis, Missouri, USA) using multiplex Luminex cytokine bead-based immunoassays (Millipore, Bedford, MA). When indicated, cells were pre-treated 30 minutes before CpG DNA stimulation with 100 μM glybenclamide (blocks ATP-sensitive K^+^ channels) (InvivoGen), high concentrations of 50 mM potassium chloride (KCl) to increase extracellular K+ concentration, 10 μM KN-62 (inhibitor of ATP induced P2X7 receptor activation) 1 μM MCC950, an inhibitor of NLRP3 inflammasome (both from Sigma Aldrich).

Supernatants were collected, 1:1 diluted in assay buffer then pre-coated color-coded microparticules with specific antibodies were added. After washing steps, a specific biotinylated antibody cocktail was added to each well. Following an incubation and washing steps to remove any unbound biotinylated antibody, streptavidin-phycoerythrin conjugate was added (Streptavidin-PE). Data were collected using the Luminex-100 system, flow-based sorting and detection analyzers (Luminex, Austin, TX).
